# Genomic and phenotypic characterization of a honey-derived *Enterococcus faecium* isolate from the Brazilian semiarid region

**DOI:** 10.1007/s11033-026-13025-w

**Published:** 2026-11-01

**Authors:** Dayane da Silva Santos, Allyson Andrade Mendonça, Maria Cecília de Lima e Silva, Marcia Maria Camargo De Morais, Danilo Elias Xavier, José Carlos da Rocha Neto, Rafael Barros De Souza, Marcos Antônio De Morais Junior

**Affiliations:** 1 Department of Genetics, Federal University of Pernambuco, Recife, Brazil; 2 Institute of Biological Sciences, University of Pernambuco, Recife, Brazil; 3 Department of Microbiology, Institute Aggeu Magalhães, FIOCRUZ, Recife, Brazil; 4 Departamento de Genética, Centro de Biociências, Universidade Federal de Pernambuco, Av. Prof. Moraes Rego, 1235, Cidade Universitária, Recife, 50670-901 Pernambuco Brazil

**Keywords:** Lactic acid bacteria, Stingless bee, Functional genomics

## Abstract

**Background:**

*Enterococcus faecium* has attracted considerable scientific interest. While some strains are recognized as opportunistic nosocomial pathogens, several non-pathogenic isolates have demonstrated beneficial properties associated with food fermentation and probiotic potential. Therefore, this study aimed to perform a functional genomic characterization of the native *E. faecium* strain EFBI58, isolated from honey in the Brazilian semiarid region.

**Materials and methods:**

*Enterococcus faecium* strain EFBI58 was sequenced using Illumina MiSeq. Bioinformatic tools were employed for comparative genomics, phylogeny, and detection of resistance, virulence, prophage, CAZyme, and bacteriocin genes. Additionally, in vitro tests assessed antimicrobial susceptibility and survival under simulated gastrointestinal conditions.

**Results:**

The EFBI58 genome presented 2,457,210 base pairs, containing 2,330 coding sequences. Phylogenomic analyses demonstrated that honey-derived isolates formed a genetically distinct group. The ANI showing belonging to clade A2 of animal-associated species. Antimicrobial resistance assessment revealed the absence of acquired genes related to clinical resistance, which was confirmed by the phenotypic susceptibility profile to the evaluated antibiotics. In addition, genes associated with important virulence factors were not identified. A prophage was 21 kb also identified in the genome. The strain EFBI58 showed survival capacity under simulated gastrointestinal conditions and the presence of stress-response proteins, demonstrating mechanisms of adaptation to adverse environments. The profile of carbohydrate-active enzymes and growth in prebiotics sugars revealed a conserved metabolic repertoire associated with the utilization of different substrates.

**Conclusions:**

Honey- and bee-associated *E. faecium* isolates possess genomic characteristics that provide a basis for future investigations involving natural antimicrobial compounds, microbial interactions, and biotechnological applications.

**Supplementary Information:**

The online version contains supplementary material available at https://doi.org/10.1007/s11033-026-13025-w.

## Introduction

The genus *Enterococcus* comprises Gram-positive, facultative anaerobic lactic acid bacteria widely distributed in diverse ecological niches, including the gastrointestinal tract of humans and animals, fermented foods, soil, water, plants, and insects [1]. Among the species of this genus, *Enterococcus faecium* has attracted considerable scientific interest due to its remarkable metabolic versatility and adaptive capacity to distinct environmental conditions. Although some strains are recognized as opportunistic nosocomial pathogens, several non-pathogenic isolates have demonstrated beneficial properties associated with food fermentation, antimicrobial production, immune modulation, and probiotic potential [2].

The ecological versatility of *E. faecium* is strongly associated with its genomic plasticity, which enables adaptation to stressful and nutrient-variable environments. This species tolerates a broad range of temperatures, pH values, osmotic pressures, and oxidative conditions, allowing its persistence in highly distinct habitats, including dairy products, fermented foods, gastrointestinal ecosystems, and insect-associated microbiota [3].The adaptive success of *E. faecium* is closely linked to horizontal gene transfer and the acquisition of mobile genetic elements, including plasmids, transposons, and genomic islands. Genomic mobility patterns can distinguish probiotic and pathogenic *E. faecium* strains, emphasizing the importance of the accessory genome in determining ecological adaptation, safety, and functional potential [4].

Honey and honeybee-associated microbiomes have recently emerged as important reservoirs of beneficial lactic acid bacteria [5]. Several bacterial species, including *E. faecium*, have been isolated from honey, bee gut, pollen, and other bee-derived products, demonstrating their ability to persist under these harsh environmental conditions. The occurrence of *E. faecium* in bee-associated environments suggests an ecological role in microbial homeostasis and host protection against pathogens [6].

The beneficial effects of bee-associated bacteria strains have attracted increasing interest in recent years. Lactic acid bacteria isolated from honeybees can inhibit pathogenic microorganisms such as *Paenibacillus larvae* through the production of organic acids, hydrogen peroxide, and bacteriocin-like compounds [7]. Consequently, bee-associated bacteria have been explored as potential probiotics for apiculture and biotechnology applications [7].

The characterization of *E. faecium* strains isolated from honeybees and honey-derived products requires careful genomic investigation, particularly because strains belonging to this species may display either probiotic or pathogenic traits depending on their genomic background [4]. In this context, comparative genomics has become an essential approach for identifying genes associated with safety, adaptation, antimicrobial production, stress tolerance, and probiotic potential. Comparative analyses involving strains isolated from insects, foods, probiotics, and clinical environments may provide valuable insights into the evolutionary diversification and functional specialization of *E. faecium* populations.

Therefore, the present study aimed to perform the genomic and phenotypic characterization of a native *Enterococcus faecium* strain isolated from honey collected in the Brazilian semiarid region. The genome of the isolate was compared with *E. faecium* strains from distinct origins, including bee-associated, probiotic, food-derived, and pathogenic isolates, in order to investigate genomic safety, functional metabolism, and probiotic potential. This study contributes to understanding the genomic and biotechnological potential of honey-associated *E. faecium* strains.

## Materials and methods

### Microorganism and culture conditions

The *Enterococcus faecium* strain EFBI58 was obtained from honey produced by the stingless bee *Scaptotrigona tubiba*. Honey sample were collected from sealed honey pots using sterile syringes and transferred to sterile 2 mL microcentrifuge tubes. Isolation was performed on modified GYC medium containing glucose (10 g/L), yeast extract (2 g/L), Tween 80 (1 g/L), ammonium citrate (2 g/L), bacteriological peptone (10 g/L), and calcium carbonate (6 g/L), supplemented with 2% (w/v) agar.

After isolation, strain EFBI58 was preserved in modified GYC medium supplemented with 15% glycerol and stored at − 80 °C. When required, the strain was reactivated in the same medium and cultivated for 24 h. Both the isolation procedure and subsequent cultivations were carried out at 37 °C under static conditions.

### DNA extraction and quality assessment

Genomic DNA was extracted from 24-h cultures according to the protocol previously described, with minor modifications [8]. Briefly, bacterial cells were harvested by centrifugation, lysed in a buffer containing Tris-HCl, EDTA, and sucrose, followed by SDS treatment. DNA was purified by two consecutive phenol–chloroform extractions and precipitated with cold isopropanol. The resulting pellet was washed with 70% ethanol, air-dried, and resuspended in sterile deionized water.

DNA concentration and purity were determined using a NanoDrop spectrophotometer. Samples presenting A260/A280 ratios of approximately 1.8 and A260/A230 ratios between 2.0 and 2.2 were considered suitable for downstream analyses. DNA integrity was verified by electrophoresis in a 0.8% agarose gel prepared in 1× TAE buffer and visualized by ethidium bromide staining. DNA samples with high purity and intact genomic bands were stored at − 20 °C until use.

### High throughput sequencing, genome assembly, and annotation

Genomic DNA extracted as described above was used for whole-genome sequencing. Paired-end genomic libraries were prepared using the Nextera XT DNA Library Preparation Kit (Illumina), purified with Agencourt AMPure XP beads (Beckman Coulter), and quantified using the Qubit dsDNA High Sensitivity Assay Kit (Invitrogen). Libraries were sequenced on an Illumina MiSeq platform using the MiSeq Reagent Kit v3 with 2 × 300 bp paired-end reads. Assembly quality metrics were calculated using QUAST v5.2.0 [9] and SeqKit v2.3.0 [10] within the Galaxy platform [11]. To determine sequencing depth and coverage breadth, quality-filtered reads were mapped back to the assembled contigs using BWA-MEM v0.7.17 [12]. Alignment metrics and coverage thresholds were computed using Samtools depth v1.15 [13] and Qualimap BAM Analysis v2.2.1 [14]. Raw sequencing reads were de novo assembled using Unicycler [15].

### Plasmid identification

The presence of plasmids was investigated using PlasmidFinder 2.1 [16]. Genome annotation was performed with Prokka v1.14.6 [17] using default parameters. Genome sequence was deposited at NCBI/GenBank database under the accession number JCAGTB010000000.

### Antimicrobial susceptibility testing and survival under simulated gastrointestinal conditions

Antimicrobial susceptibility was evaluated using the automated VITEK system according to the manufacturer’s instructions and protocols optimized for *Enterococcus* spp.

The survival of the isolate under simulated gastrointestinal conditions was evaluated as previously described. Briefly, bacterial cells were exposed to simulated gastric fluid containing 3 g/L pepsin in 0.9% saline solution adjusted to pH 2.0 and incubated at 37 °C with agitation (150 rpm) for 2 h. Subsequently, the pH was adjusted to 5.0, and 3 g/L bile salts and 1 g/L pancreatin were added to simulate the first enteric phase, followed by incubation at 37 °C and 150 rpm for 2 h. For the second enteric phase, the pH was adjusted to 7.0, and bile salts and pancreatin were added at the same concentrations, followed by a further 2 h incubation under the same conditions.

Cell viability was determined at the beginning of the assay, after the gastric phase, and at the end of the experiment by colony-forming unit (CFU) counts. Serial dilutions were plated on SYC agar and incubated at 30 °C for 48 h before enumeration.

### Comparative genomic analysis

For comparative genomic analyses, complete *Enterococcus faecium* genomes were retrieved from the NCBI database. Genomes were selected to represent isolates from different ecological niches, including hospital environments, food products, probiotic strains, and bees or bee-associated environments. Only complete genomes with available assembly and metadata information were included in the analysis. Genome-wide sequence similarity among the evaluated genomes was determined by calculating the BLASTn-based Average Nucleotide Identity (aniB) using the JSpeciesWS web server [18]. The aniB algorithm fragments the query genome sequence into consecutive 1,020 bp segments and performs reciprocal alignments against reference genomes using BLASTn [19]. Default program parameters were maintained for filtering significant alignments. The pairwise aniB value represents the mean percentage nucleotide identity calculated across all aligned genomic fragments.

The selected genomes were compared with the genome of strain EFBI58 to investigate genomic similarities and differences, as well as the distribution of genes associated with adaptation to distinct habitats and traits of biotechnological interest. Functional genome characterization and comparative analyses were conducted using the EDGAR 3.5 platform [20].

### Antibiotic resistance genes and virulence genes

Antimicrobial resistance genes were identified using the Resistance Gene Identifier (RGI) algorithm against the Comprehensive Antibiotic Resistance Database (CARD) [21]. To ensure high-confidence annotations, prediction criteria were restricted to “Perfect” (100% amino acid identity over the entire length of the reference protein) and “Strict” (matches meeting or exceeding CARD curated bitscore thresholds) algorithm options. “Lost” hits were excluded from all downstream analyses to minimize false positives and prevent the inclusion of non-functional homologues.

Putative virulence-associated genes were identified by comparison against the Virulence Factor Database (VFDB) [22]. Genome sequences were screened using the VFDB search tools and default parameters to detect homologs of known bacterial virulence factors. Identified genes were further examined based on their predicted functions and potential roles in host colonization, persistence, and pathogenicity.

### Prophage

Putative prophage regions were detected and annotated using PHIGARO v2.3.0 [23] through the Proksee platform. Open reading frames were predicted using Prodigal. Putative phage genomes were annotated using the PhageScope web server. Briefly, the FASTA sequence of the phage genome was uploaded to the platform for comprehensive structural and functional annotation. Open reading frames (ORFs) and coding sequences (CDS) were predicted using the integrated gene calling tools. Functional annotation of the predicted proteins including structural proteins, DNA packaging or replication machinery, and lysis cassette components was performed against built-in protein family databases integrated into the PhageScope workflow. PHATEST 3.0 program was used to identify the most common phage and to determine the integrity and reliability of the findings [24].

### Identification of carbohydrate-active enzymes and carbohydrate utilization assay

Carbohydrate-active enzymes (CAZymes) were identified using the dbCAN2 meta server [25], which annotates enzymes based on the CAZy classification system. Predicted protein sequences were analyzed using the default parameters of the platform to identify genes encoding glycoside hydrolases (GHs), glycosyltransferases (GTs), carbohydrate esterases (CEs), polysaccharide lyases (PLs), auxiliary activities (AAs), and carbohydrate-binding modules (CBMs). The CAZyme profile was subsequently examined to assess the carbohydrate utilization and metabolic capabilities of the isolate.

The ability of *Enterococcus faecium* EFBI58 to utilize different carbon sources was evaluated in house carbohydrate-free de Man, Rogosa, and Sharpe (MRS) broth supplemented with individual carbohydrates: glucose, fructose, lactose, maltose, fructooligosaccharides (FOS), galactooligosaccharides (GOS), inulin, trehalose, and mannose. Overnight cultures were harvested, washed, and resuspended in sterile saline solution to adjust the initial optical density at 600 nm to 0.1. Culture media supplemented with each respective carbohydrate (20 g/L, final concentration) were inoculated and incubated statically at 37 °C for 48 h. Bacterial growth was determined by measuring the final absorbance at 600 nm at the end of the 48 h incubation period using a spectrophotometer. Uninoculated MRS broth supplemented with each corresponding carbon source served as a blank control.

### Phylogenetic and structural analyses of universal stress proteins

A phylogenomic analysis based on concatenated single-copy core genes was performed using MEGA version 12 [26]. The concatenated nucleotide sequences were aligned, and a phylogenetic tree was reconstructed using the Maximum Likelihood (ML) method under the Tamura–Nei substitution model. The robustness of the inferred phylogeny was evaluated using 1,000 bootstrap replicates. The resulting tree was visualized and edited using the Interactive Tree of Life (iTOL) platform [27].

A second phylogenetic analysis was performed under MEGA version 12 [26] using the nucleotide sequences of universal stress proteins (USPs) identified in the selected genomes. The phylogenetic relationships among USP proteins were inferred using the Maximum Likelihood method with the Tamura–Nei model and 1,000 bootstrap replicates. Tree visualization and annotation were performed using iTOL [27].

### Identification of bacteriocin-encoding genes

The presence of bacteriocin-encoding genes was investigated using the BAGEL4 web server [28]. The complete genome sequence was submitted to the platform using default parameters to identify genomic regions associated with the biosynthesis of ribosomally synthesized and post-translationally modified peptides (RiPPs), bacteriocins, and other antimicrobial peptides. Predicted bacteriocin gene clusters were analyzed based on their genetic organization and similarity to previously characterized bacteriocins, with special attention given to enterocin-associated genes.

## Results

### Genome assembly features of *E. faecium* EFBI58

**Fig. 1:**
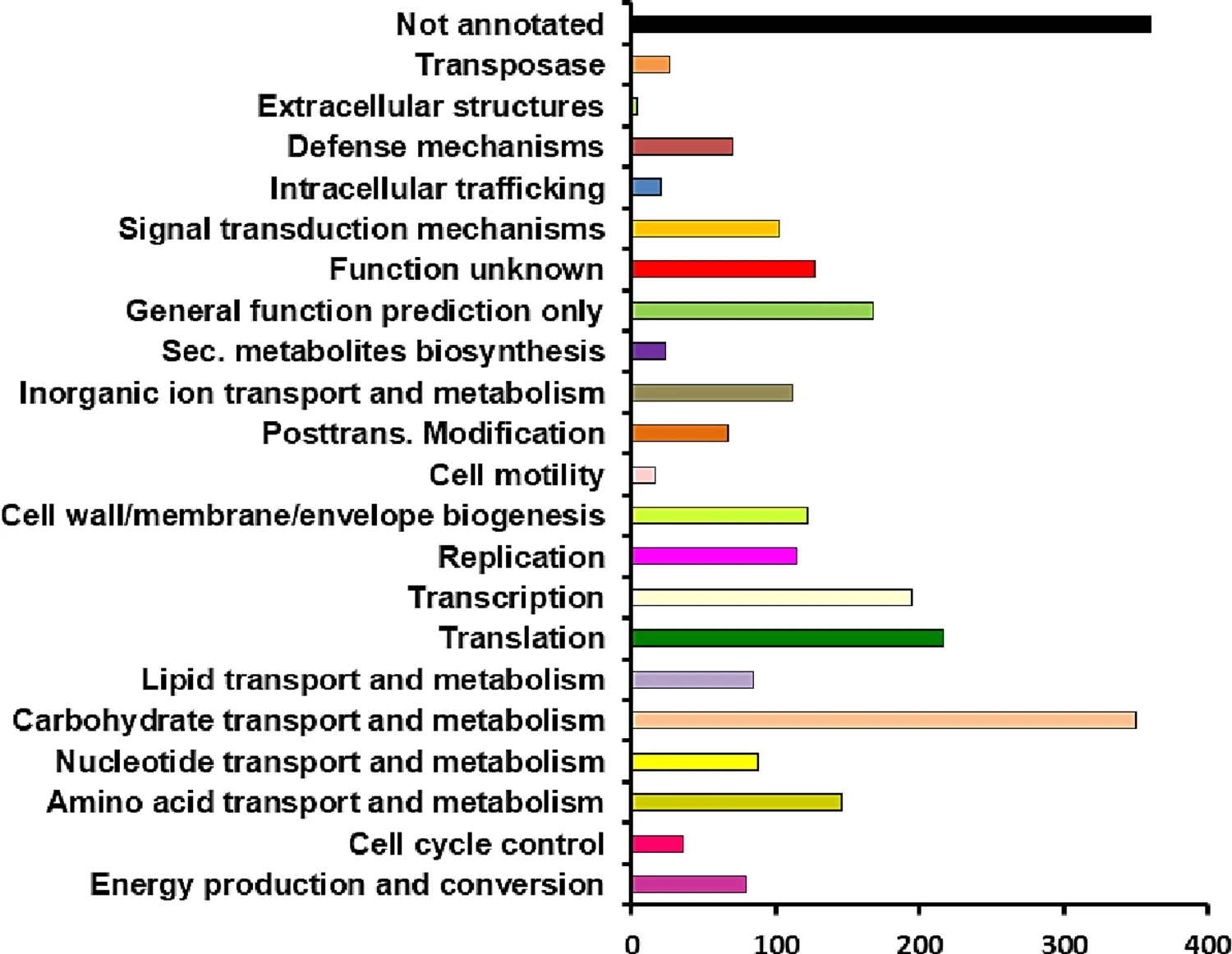
COG functional classification of complete genome of *Enterococcus.faecium* EFBI58

The genome of *Enterococcus faecium* strain EFBI58 (NCBI accession number: *JCAGTB010000000*), isolated from honey, was sequenced and assembled into 134 contigs, totaling 2,457,210 bp with an overall G + C content of 38.12% (Information on sequencing quality is in Supplementary Table S2). Genome annotation revealed the presence of 2,330 ORFs, indicating a genetic content consistent with that reported for other strains of the species. Analysis of the 16 S rRNA gene sequence revealed 99.8% similarity to *Enterococcus faecium* strains, confirming the taxonomic identification of the isolate. Furthermore, plasmid analysis using PlasmidFinder did not detect any plasmids in the genome of this strain. The complete genome was aligned with the reference strain *Enterococcus faecium* DO (TXN16) for comparative genomic analysis. The circular genome map (Supplementary Figure S1) shows the distribution of coding sequences on both the forward and reverse strands, as well as the alignment between the two genomes. Extensive coverage of homologous regions was observed across the chromosome, indicating genomic similarity of 71,5% between strain EFBI58 and the reference genome. Overall, these results demonstrate the close genetic relationship between the honey-derived *isolate E. faecium* EFBI58 and the reference strain *E. faecium* DO, supporting the taxonomic identification Fig. 1.

### Comparative genomic relationships among honey, food, probiotic, and hospital-associated *E. faecium* isolates

**Fig. 2:**
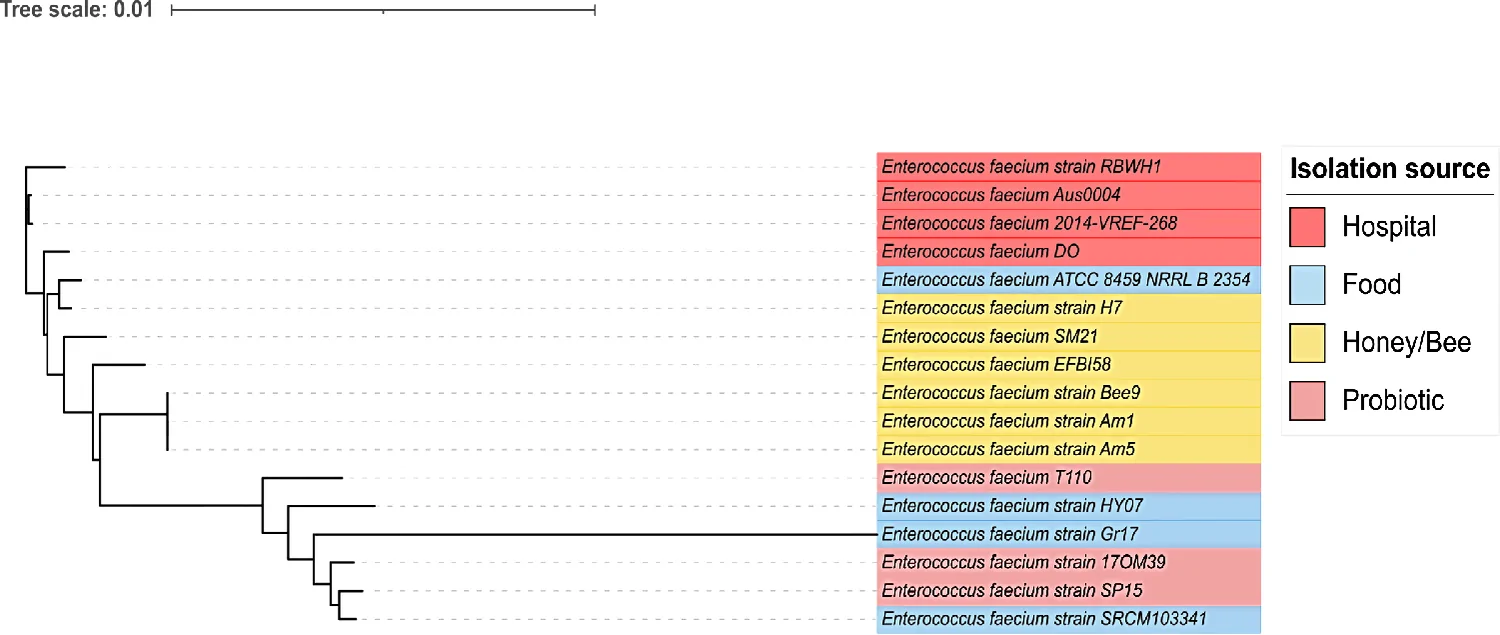
Phylogenetic Maximum Likelihood analysis of *Enterococcus faecium* strains from different origins. Colors indicate the source of isolation: probiotic (pink), honey (yellow), food (blue), and hospital (red)

Comparative genomic analysis including 17 *E. faecium* completes genomes (Supplementary table S1) isolated from honey, food, probiotic, and hospital environments revealed a clear clustering according to ecological origin (Fig. 2). Honey-derived isolates (EFBI58, H7, Bee9, SM21, Am1, and Am5) formed a closely related genomic cluster, indicating a high degree of genomic conservation among strains associated with stingless bee honey.

Food-derived and probiotic strains occupied neighboring positions, whereas most hospital-associated isolates formed separate branches, reflecting the genomic diversification associated with adaptation to clinical environments. Although some overlap between environmental and clinical isolates was observed, the clustering pattern clearly demonstrated that honey-associated strains constitute a distinct evolutionary group sharing greater genomic similarity with food-associated isolates than with multidrug-resistant hospital lineages.

Phylogenomic analysis based on Average Nucleotide Identity (ANI) revealed high sequence similarity between *Enterococcus faecium* EFBI58 and strains isolated from honey and bees, exhibiting ANI values greater than 99%. Furthermore, strain EFBI58 shared 98.9% ANI with a swine-derived reference strain belonging to Clade A2. In contrast, lower genomic similarity was observed when compared to pathogenic *E. faecium* strain Aus004 (98.8%) (Supplementary Table S3). Notably, pairwise ANI values dropped below the 95% species delineation threshold when compared with food-derived and probiotic strains belonging to Clade B (*Enterococcus lactis*), confirming that strain EFBI58 belongs strictly to *E. faecium sensu stricto* (Clade A).

A pangenome analysis was performed to evaluate gene conservation and genomic diversity among the 17 *Enterococcus faecium* genomes (Supplementary Figure S2). The pan genome consists of 5198 CDS with *E.faecium* EFBI58 used as reference. The UpSet plot revealed a large conserved core genome, with 1,595 orthologous gene clusters shared by all strains, representing the major component of the species genomic repertoire.

In addition to the core genome, numerous accessory gene clusters were identified, with the largest shared accessory groups, each distributed among different subsets of strains. Several smaller gene clusters, ranging from approximately 17 to 88 genes, were also observed, highlighting the extensive variability of the accessory genome. The horizontal bar plot showed that all genomes contained a comparable number of coding sequences (CDSs), indicating similar genome sizes despite differences in gene content. Overall, the results demonstrate that *E. faecium* possesses a highly conserved core genome together with a diverse accessory genome, reflecting ongoing gene gain and loss during evolution. The highest number of unique genes was observed in the pathogenic strains (187 ORFs in strain Aus0004), whereas the lowest was found in the honey-derived strain Am5 (19 unique ORFs). Strain EFBI58, isolated from Brazilian honey, exhibited 88 unique ORFs, notably including a vitamin B12-dependent ribonucleotide reductase and a corrinoid adenosyltransferase. The presence of these genes suggests an enhanced capacity for cobalamin-dependent metabolism, which may contribute to metabolic flexibility and adaptation to nutrient-variable environments. Interestingly, these unique genes displayed GC contents of 45% and 34%, respectively, which differ from the overall genomic GC content of *E. faecium* EFBI58 (38.1%). This variation in GC composition may indicate the acquisition of these genes through horizontal gene transfer events.

### Phenotypic and genomic assessment of antimicrobial resistance

Antimicrobial susceptibility testing demonstrated that EFBI58 was susceptible to all antibiotics evaluated (Table 1). Low minimum inhibitory concentration (MIC) values were observed for ampicillin, teicoplanin, tigecycline, vancomycin, linezolid, and levofloxacin. In addition, no high-level resistance to streptomycin or gentamicin was detected, indicating the absence of clinically relevant acquired resistance.

**Table 1:** Minimum Inhibitory Concentration (MIC) values and antimicrobial susceptibility profile of *Enterococcus faecium* strain EFBI58

Antibiotic	Drug class	MIC	Interpretation*
Ampicillin	β-lactams	≤ 2 µg/mL	Susceptible
Streptomycin	Aminoglycoside	SIN-S	Susceptible
Gentamicin	Aminoglycoside	SIN-S	Susceptible
Levofloxacin	Fluoroquinolone	4 µg/mL	Susceptible
Linezolid	Oxazolidinone	2 µg/mL	Susceptible
Teicoplanin	Glycopeptide	≤ 0.5 µg/mL	Susceptible
Tigecycline	Glycylcycline	≤ 0.12 µg/mL	Susceptible
Vancomycin	Glycopeptide	≤ 0.5 µg/mL	Susceptible

*SIN-S: absence of high-level aminoglycoside resistance

**Fig. 3:**
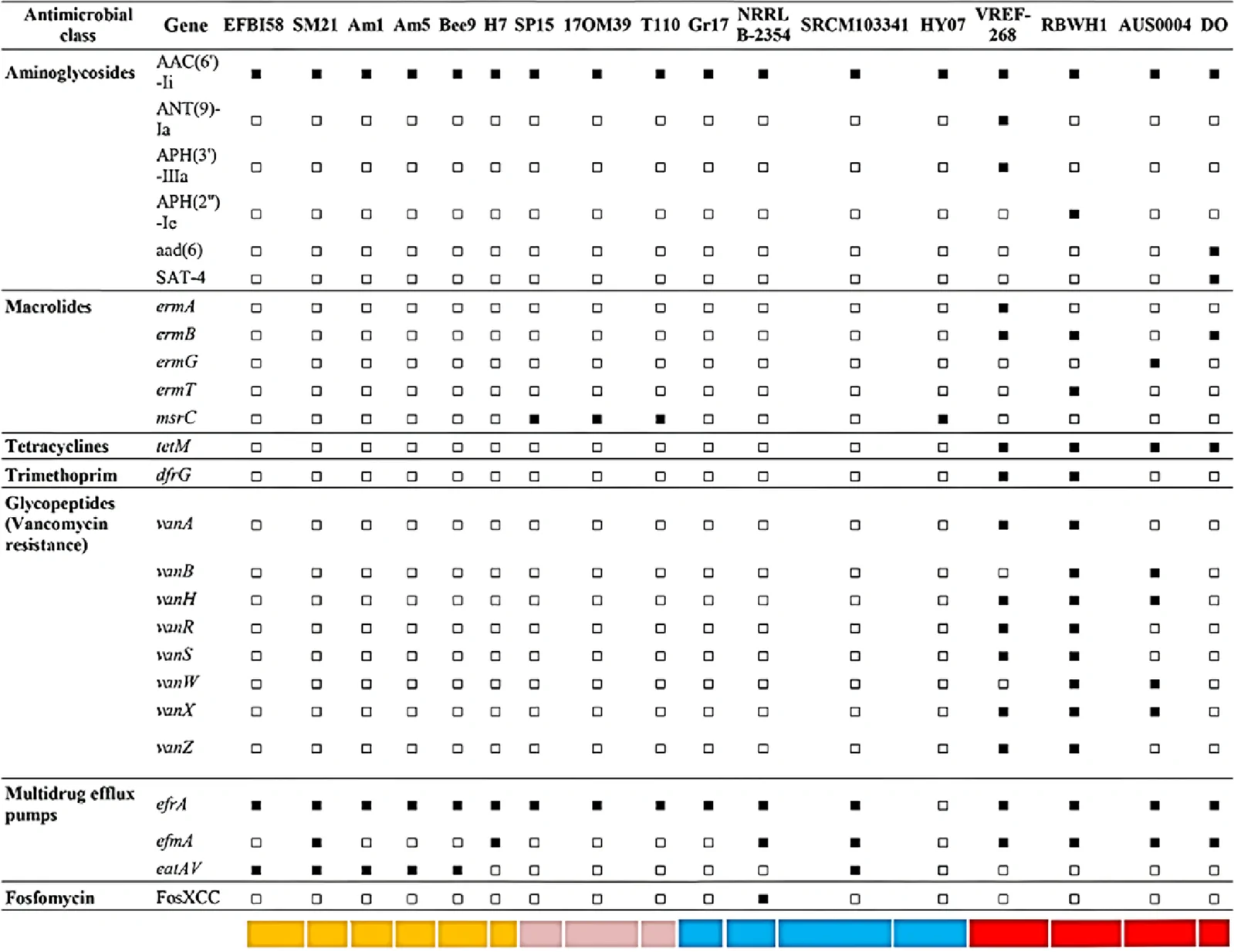
Distribution of antimicrobial resistance genes among *Enterococcus faecium* strains from different origins. Rows represent resistance genes and columns represent individual strains. Colored cells indicate the presence of a gene, whereas blank cells indicate its absence. The color bar at the bottom identifies strain origin: honey (yellow), Probiotic (pink), Food (blue), and hospital (red)

Comparative resistome analysis supported the phenotypic results (Fig. 3). Honey-derived isolates exhibited a markedly reduced repertoire of antimicrobial resistance genes compared with hospital-associated strains. EFBI58 lacked acquired genes conferring resistance to glycopeptides, tetracyclines, and aminoglycosides. Only intrinsic resistance determinants, including *efrA*,* AAC(6’)-li* and *eatA*(V) with alignment values exceeding 98%, were consistently detected among honey isolates. In contrast, hospital-associated genomes contained multiple acquired resistance genes, particularly the complete van operon (*vanA*,* vanH*,* vanR*,* vanS*,* vanX* and *vanW*), together with aminoglycoside- and macrolide-resistance determinants. The vancomycin-resistant strain VREF-268 displayed the largest resistome among all genomes analyzed.

Importantly, complete agreement was observed between phenotypic susceptibility testing and genomic prediction for EFBI58. The absence of clinically relevant resistance genes corroborated the fully susceptible antimicrobial profile, supporting the classification of EFBI58 as a non-clinical environmental isolate with a favorable biosafety profile.

### Virulence profile and genomic safety

**Fig. 4:**
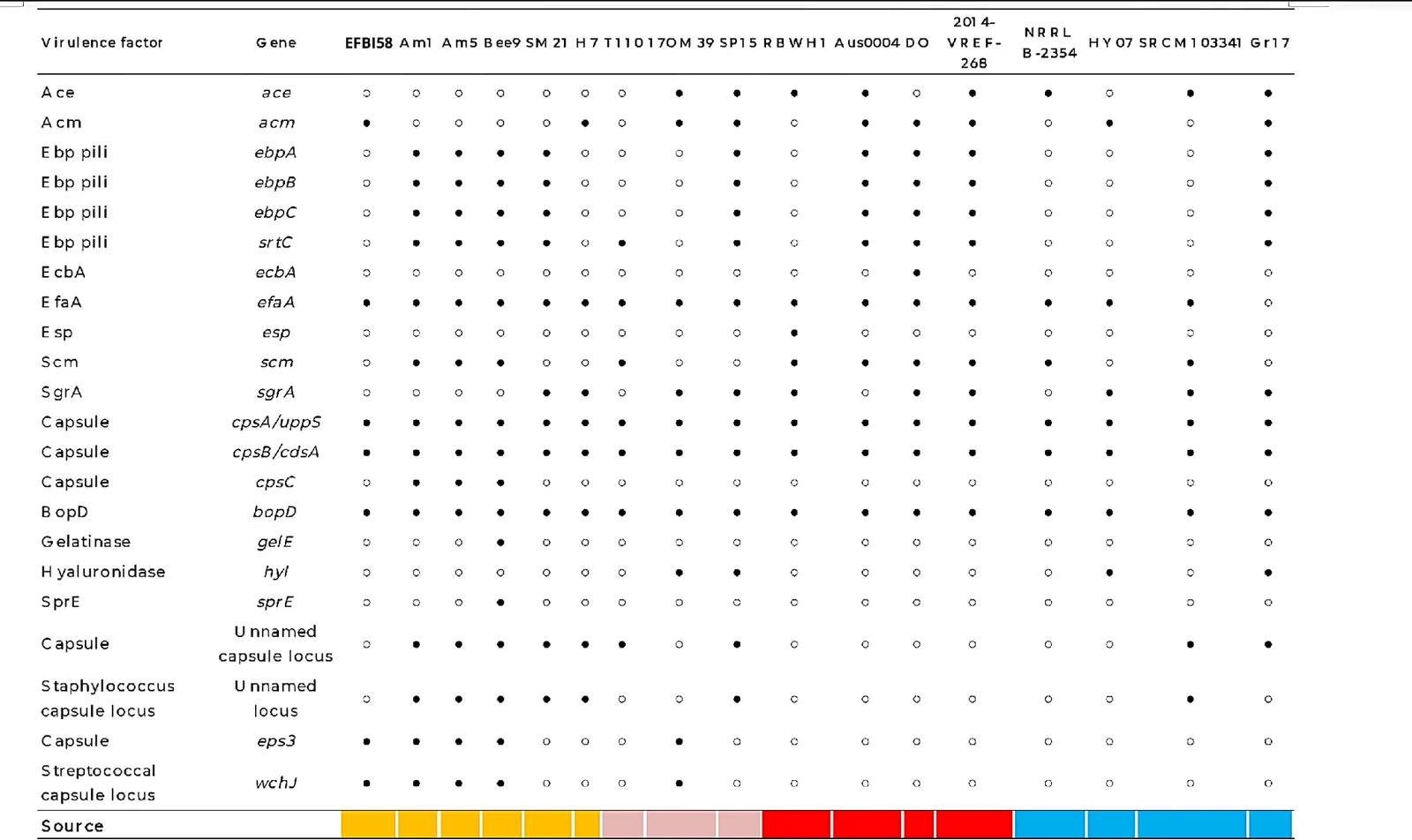
Distribution of virulence-associated genes among *Enterococcus faecium* isolates from honey, probiotic, food, and hospital sources. Rows represent virulence genes grouped according to their functional categories (capsule, adhesion, biofilm formation, toxin production, and immune evasion), while columns correspond to individual isolates. Blue and pink cells indicate the presence and absence of genes, respectively. Isolates are color-coded according to their source: honey (yellow), probiotic (pink), food (blue), and hospital (red)

Comparative analysis of virulence-associated genes revealed substantial variation in adhesion- and capsule-related genes among the analyzed genomes, whereas genes encoding major toxins were uniformly absent (Fig. 4). Capsule-associated genes (*cpsA/uppS* and *cpsB/cdsA*) were highly conserved across all genomes, while the remaining capsule biosynthesis genes showed variable distributions. Among adhesion-associated genes, *acm*,* scm*,* sgrA*,* ecbA*, and *esp* exhibited heterogeneous occurrence independent of isolate origin. Regarding adhesion factors, EFBI58 carried the collagen-binding adhesin gene *acm* but lacked the *scm* gene, which encodes a second collagen-binding microbial surface component.

Notably, genes encoding cytolysin production (*cylA*,* cylB*,* cylL*,* cylM*,* cylR1*, and *cylR2*) were absent from all analyzed genomes. Likewise, *gelE*, *fsrB*, and *fsrC* were either absent. Overall, honey-derived isolates did not exhibit an enrichment of virulence-associated determinants compared with food or probiotic strains. Instead, they displayed genomic profiles characterized by the conservation of housekeeping colonization factors and the absence of major pathogenicity-associated genes, reinforcing their favorable safety profile.

Accessory capsule-associated genes displayed greater variability. The Staphylococcal capsule locus was detected in all honey-derived isolates (Am1, Am5, and Bee9) but was absent from EFBI58, distinguishing this strain from the other isolates obtained from honey. In contrast, the capsule-associated glycosyltransferase gene *wchJ* was identified in EFBI58, Am1, Am5, Bee9, and SP15, whereas it was absent from the remaining genomes. The *eps3* gene, involved in exopolysaccharide biosynthesis, was present in EFBI58, Am1, Am5, Bee9, T110, and SP15, but absent from SM21, H7, and 17OM39. Overall, EFBI58 shared the conserved core capsule biosynthesis genes with the other non-pathogenic isolates but exhibited a distinct accessory capsule gene profile.

### Stress tolerance: integration of gastrointestinal survival and stress-response proteins

The ability of EFBI58 to tolerate gastrointestinal stress was evaluated using an in vitro digestion model. Cell viability progressively decreased throughout exposure to simulated gastric and intestinal fluids, from approximately 9.5 log CFU mL − 1 to 2.3 log CFU mL − 1 after completion of the assay (Supplementary Figure S3). Despite this reduction, viable cells remained detectable throughout the experiment, demonstrating the capacity of EFBI58 to survive sequential acid and bile stresses.

**Fig. 5:**
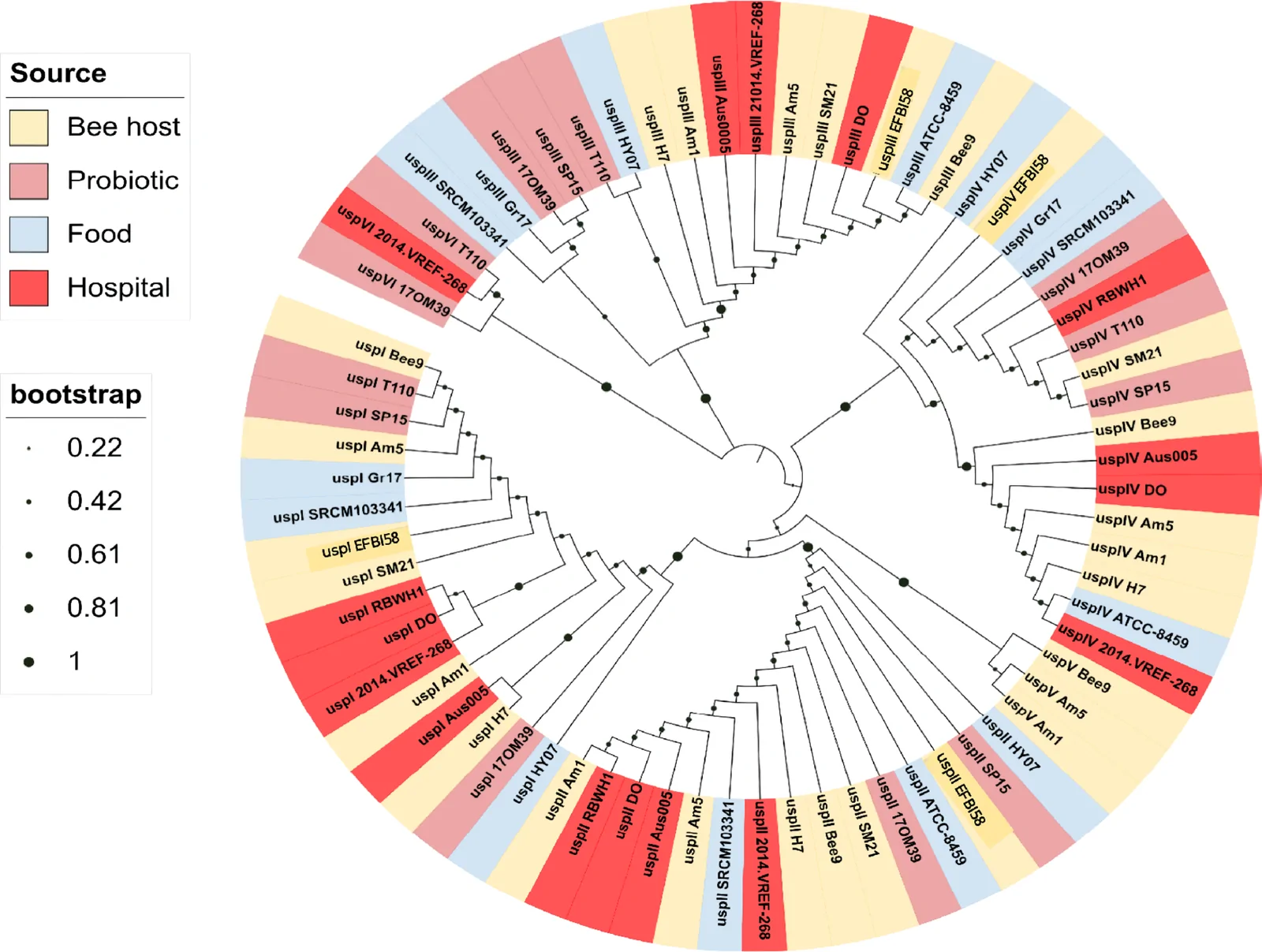
Maximum Likelihood phylogenetic tree of genes coding Universal Stress Proteins (USPs) from *Enterococcus faecium* isolates obtained from bee hosts, food, probiotic, and hospital sources. Six major USP clades (USPI–USPVI) were identified. Branch support is indicated by node size according to bootstrap values, and representative predicted three-dimensional structures are displayed for each clade. Isolate origins are represented by colored bars surrounding the tree

To investigate the genomic basis underlying stress adaptation, universal stress proteins (Usps) were comparatively analyzed across the 17 genomes. Phylogenetic reconstruction identified six major USP clades (USPI–USPVI) (Fig. 5). Most Usp families contained proteins from isolates of multiple ecological origins. However, the UspV clade consisted exclusively of honey-derived isolates.

Structural prediction demonstrated that all representative USP proteins shared highly conserved three-dimensional architectures despite sequence divergence, indicating functional conservation throughout evolutionary diversification. The combination of phenotypic gastrointestinal tolerance and the presence of diversified stress-response proteins supports the remarkable adaptive capacity of honey-associated *E. faecium* isolates under harsh environmental conditions.

### Carbohydrate utilization and CAZyme repertoire

**Fig. 6:**
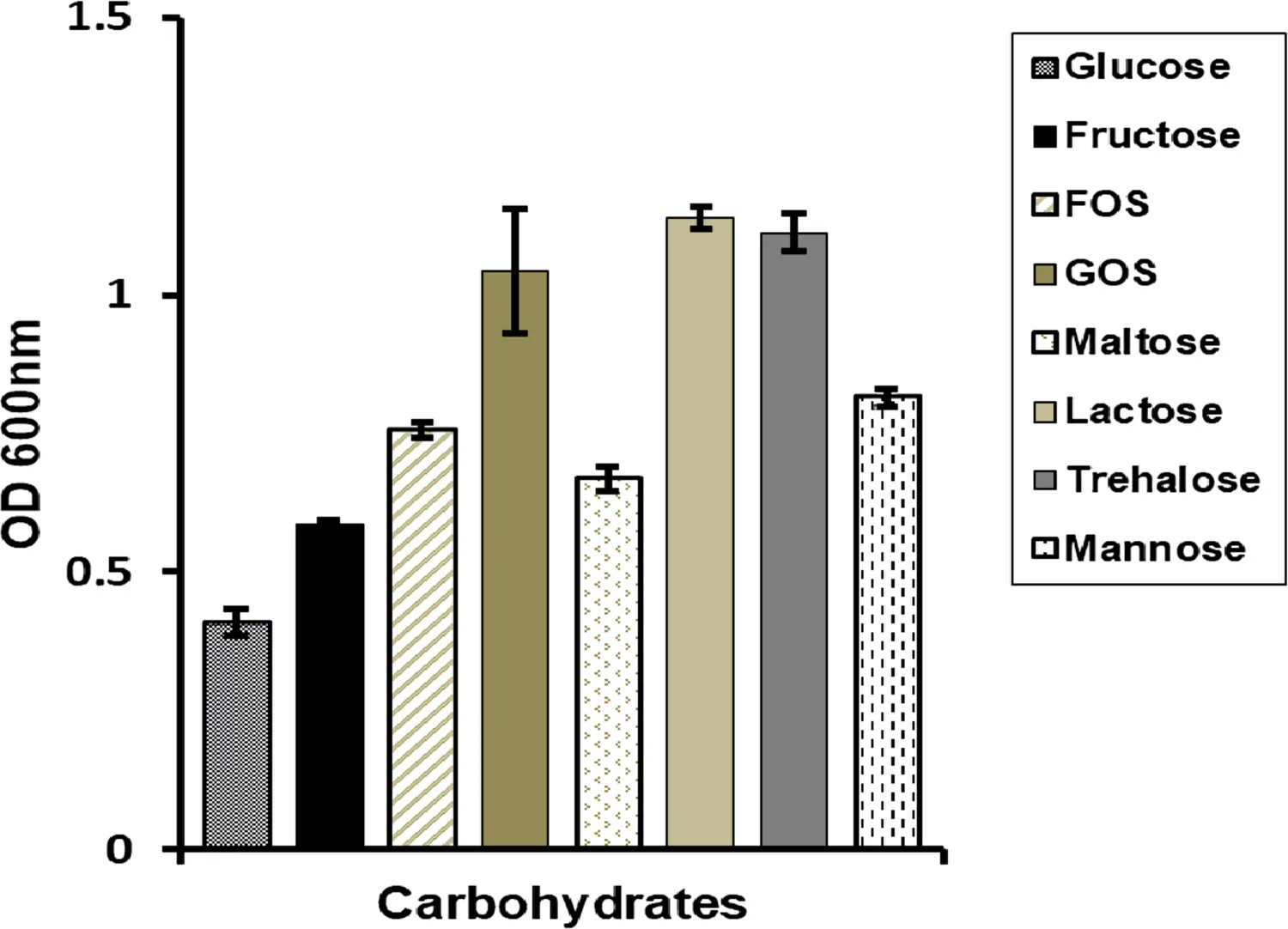
Carbohydrate utilization profile of *Enterococcus faecium* strain EFBI58. The growth response of *E. faecium* EFBI58 was evaluated in the presence of different carbohydrates as sole carbon sources. Bacterial growth was monitored by measuring optical density at 600 nm (OD600). The tested carbohydrates included glucose, fructose, fructooligosaccharides (FOS), galactooligosaccharides (GOS), maltose, lactose, trehalose, and mannose. Bars represent the mean values ± standard deviation of the experiments

The ability of *Enterococcus faecium* EFBI58 to utilize different carbohydrates was evaluated by measuring cell growth (OD600) after incubation (Fig. 6). The strain exhibited distinct growth patterns depending on the carbon source. The highest optical density values were observed in media supplemented with lactose, trehalose, and galactooligosaccharides (GOS), indicating that these substrates supported the most efficient growth. Intermediate growth was observed in the presence of mannose, fructooligosaccharides (FOS), maltose, and fructose, whereas glucose supported the lowest detectable growth. In contrast, no growth was detected when inulin or xylose was provided as the sole carbon source, indicating that EFBI58 is unable to metabolize these substrates under the conditions tested. Overall, the results demonstrate that EFBI58 efficiently utilizes several mono-, di-, and oligosaccharides, while exhibiting a restricted carbohydrate utilization profile for inulin and xylose.

**Fig. 7:**
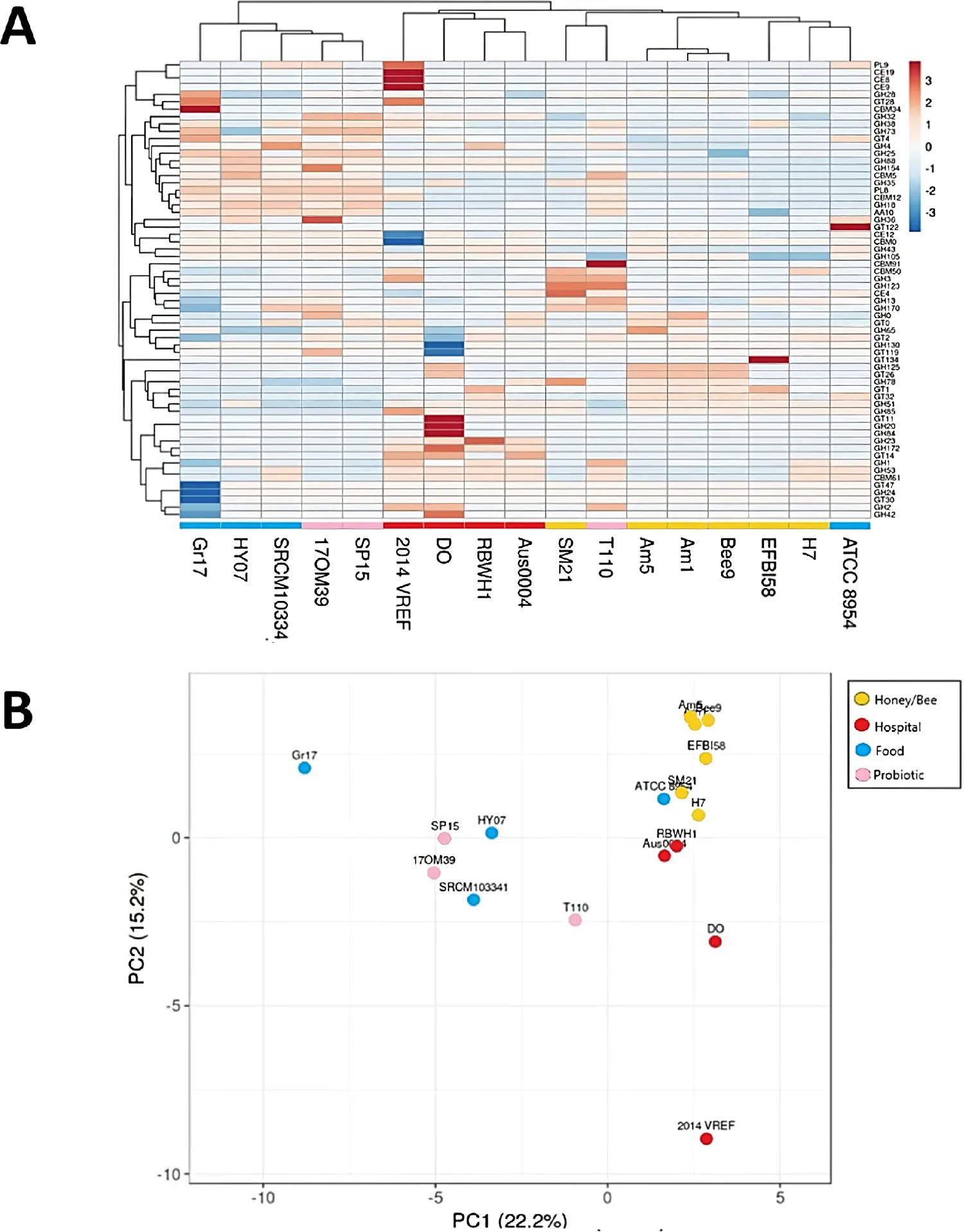
Comparative analysis of carbohydrate-active enzymes (CAZymes) in *Enterococcus faecium* genomes. **A**) The heatmap represents the distribution and abundance of CAZyme families identified among *E. faecium* strains, with hierarchical clustering based on similarity of CAZyme profiles. Rows correspond to CAZyme families and columns represent the analyzed genomes. The color scale indicates the relative abundance of CAZyme genes across strains.** B**) The principal component analysis (PCA) plot shows the genomic variation among strains based on their CAZyme repertoires, highlighting the relationship between isolates from different sources (food, probiotic, hospital, and honeybee). The position of strain EFBI58 indicates its CAZyme profile in comparison with other *E. faecium* genomes

To further investigate the carbohydrate metabolic potential of *E.faecium* EFBI58 and related isolates, carbohydrate-active enzymes (CAZymes) were identified and compared among honey, food, probiotic, and hospital-associated *E. faecium* genomes (Fig. 7A). All analyzed genomes exhibited highly conserved CAZyme repertoires dominated by glycosyltransferases (GTs) and glycoside hydrolases (GHs), whereas carbohydrate esterases (CEs), carbohydrate-binding modules (CBMs), and polysaccharide lyases (PLs) were represented by fewer families.

The most abundant glycosyltransferase families included GT2 and GT4, which are involved in the biosynthesis of cell wall polysaccharides and glycoconjugates. Among glycoside hydrolases, members of the GH1, GH13, GH36, GH42, GH65, GH73, GH92, GH170, and GH172 families were consistently identified across the analyzed genomes, reflecting the ability of *E. faecium* to hydrolyze a broad range of oligo- and polysaccharides. In addition, carbohydrate esterases belonging to the CE11 family and carbohydrate-binding modules represented by CBM50 were conserved among most isolates, suggesting maintenance of essential carbohydrate-processing functions.

In addition, a principal component analysis (PCA) was performed using the abundance of CAZyme families identified in each genome (Fig. 7B). The first two principal components explained 37.4% of the total variation, with PC1 accounting for 22.2% and PC2 for 15.2%. The PCA revealed moderate clustering of strains according to their CAZyme repertoires rather than a strict separation based on isolation source. The honey/bee isolates EFBI58, Am5, Bee9, SM21, and H7 clustered closely in the positive region of PC1, indicating highly similar CAZyme profiles. Among them, EFBI58 grouped near Am5 and Bee9, suggesting conservation of carbohydrate utilization potential within these bee-associated strains.

Hospital isolates showed greater dispersion across the ordination space. While Aus0004 and RBWH1 clustered close to the honey-associated strains, DO and particularly 2014-VREF-268 were clearly separated along PC2, indicating greater divergence in their CAZyme repertoires. Food-associated isolates also exhibited heterogeneous distribution, with HY07 and SRCM103341 positioned close to the central cluster, whereas Gr17 was clearly separated along the negative axis of PC1. Probiotic strains displayed intermediate positions, with SP15 and 170M39 clustering near the food isolates and T110 occupying a distinct position.

Overall, these results indicate that the CAZyme repertoire is largely conserved among several *E. faecium* strains, particularly those associated with honey and bees, whereas greater variability is observed among hospital-derived isolates.

### Prophage

A 21.9-kb questionable prophage (score 70) region belonging to the Siphoviridae family was identified in the genome of *Enterococcus faecium* EFBI58, spanning positions 152,599 to 174,540 (Supplementary Figure S4). Genome annotation identified 19 open reading frames (ORFs) encoding proteins involved in phage structure, DNA replication, genome packaging, integration, and host infection, supporting the classification of this region as an intact temperate prophage. The predicted proteins include terminase subunits, a portal protein, capsid proteins, integrases, endonucleases, and several hypothetical proteins. Additionally, four hypothetical ORFs of bacterial origin were detected within the prophage region. The prophage exhibited a GC content of 36.96%. The most common prophage was the *Lactococcus* prophage bIL311, found in *Lactococcus lactis* IL1403. It also showed similarity to the *Enterococcus* phage vB_EfaS_AL2 from *Enterococcus faecalis*.

Comparative analyses indicated that this prophage shares limited sequence similarity with prophages previously reported in *E. faecium* isolates associated with bees (Supplementary Figure S5). This finding suggests that the prophage may represent a distinct lineage or a highly divergent mobile genetic element. The abundance of hypothetical proteins and uncharacterized open reading frames further supports the possibility that this prophage contains genetic features that are currently underrepresented in public databases.

### Bacteriocin-related genes

**Fig. 8:**
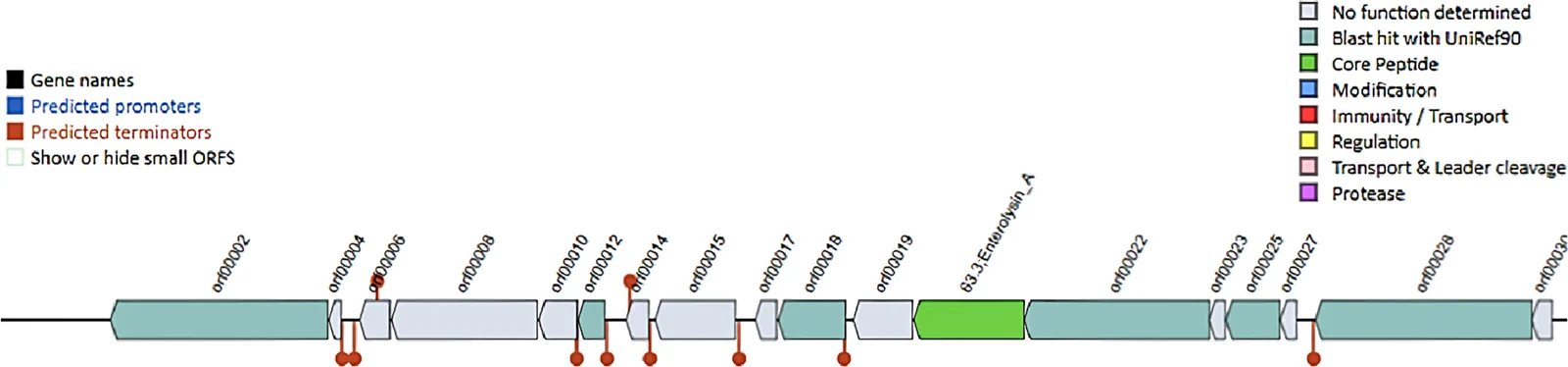
Genetic organization of the enterolysin A gene Identified in *Enterococcus faecium* EFBI58. The schematic representation shows the predicted coding sequences (CDSs), promoter regions, and terminator sites within the enterolysin A-associated locus. Genes are color-coded according to their predicted functional categories, including structural and accessory proteins, transport-related proteins, regulatory elements, and hypothetical proteins

Genome mining for bacteriocin-associated genes revealed that *E.faecium* EFBI58 harbors the gene encoding enterolysin A (*entA*), whereas no genes encoding classical enterocins were identified (Fig. 8). Enterolysin A is a cell wall-degrading bacteriolysin that differs from conventional enterocins in both structure and mode of action. This finding suggests that EFBI58 may rely on a lytic antimicrobial mechanism rather than on the production of small pore-forming bacteriocins.

## Discussion

*Enterococcus faecium* is lactic acid bacteria frequently associated with clinical environments due to the emergence of multidrug-resistant lineages. However, comparative studies demonstrate that environmental populations exhibit high genomic diversity and harbor strains with safety and functional profiles distinct from hospital-associated isolates [29]. In this context, the isolation of EFBI58 of honey highlights natural niches as reservoirs of non-pathogenic and functionally versatile microorganisms [4]. The association of *Enterococcus* species with bee ecosystems is well documented. Members of this genus are common components of honeybee (*Apis mellifera*) colonies and associated products, having been reported in adult bees since the early 1970s [5, 6].

The carbohydrate utilization profile of *Enterococcus faecium* EFBI58 revealed variations in final biomass yield, with higher cell densities recorded in the presence of disaccharides (lactose and trehalose) and oligosaccharides compared to monosaccharides (glucose and fructose).The higher biomass achieved in trehalose, relative to glucose and fructose, may reflect an ecological adaptation to the isolate’s origin, given that trehalose is a major sugar component in insect hemolymph and bee-associated matrices [30]. Furthermore, sustained growth on galactooligosaccharides, fructooligosaccharides and mannose underscores the efficiency of transport systems and glycoside hydrolases in *E. faecium EFBI58* for fermenting diverse substrates, conferring notable nutritional versatility, including prebiotic sugars [30].

The genomic size observed for EFBI58 is within the typical range described for *E. faecium*, a species characterized by compact genomes, high genomic plasticity, and remarkable ecological adaptability [3]. Importantly, the absence of plasmids in EFBI58 suggests a low risk of horizontal gene transfer carrying undesirable traits, which is a critical safety parameter for biotechnological strains, unlike clinical lineages where mobile genetic elements frequently spread antimicrobial resistance and virulence determinants [2].

Phylogenomic analysis demonstrated that honey-derived isolates formed a genetically related cluster, showing greater similarity to food and environmental lineages than to hospital-associated isolates. This pattern agrees with the established population structure of *E. faecium*, which is divided into three distinct clades: Clade A1, associated with hospital environments and multidrug-resistant clinical infections; Clade A2, predominantly associated with livestock and domestic animals; and Clade B, representing community-associated strains commonly isolated from healthy individuals and natural environments, which rarely cause disease and is being reclassified as *Enterococcus lactis* [31]. The genomic differentiation observed in EFBI58 reinforces its ANI with non-clinical lineages (Clade A2 derivatives), confirming that natural niches select for bacterial groups adapted to specific physiological conditions rather than clinical pathogenicity. Because Clade A2 is closely related to lineages adapted to animal hosts, the resistome (CARD) and virulence (VFDB) screening performed for strain EFBI58 becomes a critical step to certify its biological safety, ensuring the absence of classical multi-drug resistance determinants prior to any functional or apicultural application [4].

The genomic resistome of *Enterococcus faecium* EFB58 is strictly intrinsic and non-MDR, featuring aac(6’)-Ii and eatAV. The presence of aac(6’)-Ii and eatAV represents the species-specific core resistome, mediating baseline low-level resistance to select aminoglycosides and lincosamides/streptogramins via target protection mechanisms [32]. Although automated prediction tools initially flagged a putative *vanY* gene, it exhibited a sequence identity of only 34.48%, well below standard detection thresholds (> 80%), representing a bioinformatic artifact stemming from conserved catalytic domains in endogenous D, D-carboxypeptidases rather than a functional resistance gene [33]. This, combined with the complete absence of an intact, functional *van* operon (such as *vanA* or *vanB* clusters), confirms that EFB58 lacks a Vancomycin-Resistant *Enterococcus* (VRE) phenotype [34]. Furthermore, the total lack of acquired resistance determinants (e.g., *erm(B)*,* tet(M)*,* optrA*) underscores that strain EFB58 has not been subjected to clinical antimicrobial selection pressure or mobile genetic element acquisition [34]. Comparative resistome analysis confirmed that while hospital strains carry diverse determinants against glycopeptides, aminoglycosides, and macrolides due to clinical selective pressure, honey-derived isolates lack acquired clinically relevant resistance genes, confirming their environmental safety status.

Virulence profiling further supported EFBI58’s non-pathogenic nature. Key genes responsible for cytolysin production (c*ylA*,* cylB*,* cylL*,* cylM*,* and cylR*), which represent the primary toxin driving enterococcal pathogenicity [2], were absent. Furthermore, genes involved in colonization and biofilm formation were distributed variably, without indicating a pathogenic phenotype. This combination of high functional diversity and low virulence highlights EFBI58 as a safe candidate for bioprocess development.

From a functional perspective, EFBI58 demonstrated high viability under simulated gastrointestinal conditions (acidic pH and bile salts), revealing stress tolerance mechanisms indicating that this strain tolerates passage through the gastrointestinal tract, which is one of the fundamental tests for a bacterium to be considered probiotic [2]. In this case, the EFBI58 strain demonstrated potential for future studies on the development of probiotic strains for bees. In addition, comparative analysis revealed that specific universal stress proteins (USPs) were uniquely present in honey-derived *E. faecium* strains, distinguishing them from other lineages. USPs are conserved bacterial proteins associated with cell survival under adverse conditions, such as osmotic fluctuations, nutrient depletion, and oxidative stress [35]. In *E. faecium*, a universal stress protein gene (*usp*) was experimentally confirmed to be essential for bacterial survival during prolonged starvation, highlighting its role as a key determinant of environmental robustness [36]. The exclusive presence of specific USP determinants in honey-isolated strains suggests that these strains retained the USP protein despite selective pressures, likely because it confers a fitness advantage in their environment of origin [35].

Notably, genomic analysis revealed specific metabolic genes uniquely present in strain EFBI58, including a vitamin B12-dependent ribonucleotide reductase and a corrinoid adenosyltransferase. Ribonucleotide reductases are crucial for catalytic conversion of ribonucleotides to deoxyribonucleotides during DNA synthesis and repair, and the reliance on cobalamin (vitamin B12) cofactor machinery suggests an enhanced capability for DNA replication and repair under stressful or anaerobic/microaerophilic conditions [37]. Corrinoid adenosyltransferases further play an indispensable role in cobalamin cofactor activation and cofactor salvage pathways [38]. The exclusive presence of these enzymatic systems in EFBI58 highlights a tailored metabolic adaptation for enhanced nucleic acid biosynthesis, cofactor utilization, and stress endurance in nutrient-variable niches, underscoring their potential utility in targeted biotechnological applications.

Beyond stress resistance and metabolic versatility, the strain harbors encoding regions for bacteriocins (enterocins). These ribosomally synthesized antimicrobial peptides represent a major biotechnological asset, conferring ecological competitiveness and offering potential applications as novel natural bio-preservatives in food systems or biotherapeutic agents against foodborne pathogens [39].

Finally, genomic analysis identified prophage regions within EFBI58 that lack known virulence or resistance genes. Rather than increasing pathogenicity, these bacteriophages appear to contribute to host fitness, evolutionary adaptation, and metabolic diversification in selective environments [40]. Prophage similarity to *Lactococcus* phage bIL311 and *E. faecalis* phage vB_EfaS_AL2 alongside *E. faecium* phage EFBI58 reflects the mosaic architecture and horizontal gene transfer (HGT) typical of *Lactobacillales* phages [41]. Automated database matches to *Lactococcus* occur due to highly conserved functional modules (e.g., integrases, tail proteins) shared among lactic acid bacteria occupying common ecological niches [42]. Overall, *E. faecium* EFBI58 represents a non-clinical strain that combines stress tolerance, a safe resistome/virulome profile, robust carbohydrate metabolism, unique cobalamin-dependent metabolic capabilities, and bacteriocinogenic potential. These findings establish honeybee-associated environments as rich sources of functional microbial diversity and position EFBI58 as a promising candidate for probiotic formulation, food bioconservation, and biotechnological processing.

## Conclusion

Genomic and phenotypic evaluations revealed that *E.faecium* EFBI58 isolated from honey presents a favorable profile, including the absence of plasmids, low occurrence of acquired antimicrobial resistance determinants, and absence of important virulence factors, including genes associated with cytolysin production. These findings distinguish environmental isolates from clinical *E. faecium* lineages commonly associated with resistance and pathogenicity. Furthermore, the ability of *E.faecium* EFBI58 to survive under simulated gastrointestinal conditions and the conservation of carbohydrate metabolism-related enzymes demonstrate important mechanisms for biotechnological aplications. The identification of bacteriocin-associated genes and phage-related elements highlights the genetic complexity of these strains and their role in natural microbial interactions. Overall, these results emphasize honey and bee-associated environments as important reservoirs of microbial diversity and demonstrate that *E. faecium* isolates from these niches possess genomic characteristics of biotechnological interest. These findings provide a basis for future investigations involving natural antimicrobial compounds, microorganism interactions, and biotechnological applications.

## Supplementary Information

Below is the link to the electronic supplementary material.

Supplementary Material 1

Supplementary Material 2

## Data Availability

Genome sequence was deposited at NCBI/GenBank database under the accession number JCAGTB010000000.
