# Supplementary material for: Genomic and phenotypic characterization of a honey-derived Enterococcus faecium isolate from the Brazilian semiarid region
Source: Mol Biol Rep. 2026 Nov 1;53(1):1875. doi: 10.1007/s11033-026-13025-w (PMC13724667; doi:10.1007/s11033-026-13025-w)
Supplement: Supplementary file 1 [file 11033_2026_13025_MOESM1_ESM.pdf]

Comparative Genomics Reveals Environmental Adaptation and Biotechnological Potential of a Honey-Derived Enterococcus faecium from the Brazilian Semiarid Region

Santos et al.

Supplementary Table S1. Bacterial strains used in this study, including GenBank accession numbers of their genome sequences.

Strain	Source	Country	Accession number*	Genome length (pb)	Reference
EFBI58	Honey	Brazil	JCAGTB010000000	2,457,210	This study
Am1	Bee gut – larvae	Egypt	JAHLTJ000000000	2,732,750	Zaghloul et al. 2022
Bee9	Bee gut – Adult	Egypt	JAHLXG000000000	2,734,007	Zaghloul et al. 2022
SM21	Bee gut	Argentina	NZSDXT00000000	2,544,244	Audisio, 2019
H7	Bee gut	China	CP083179	2,529,646	Du et al. 2021
Am5	Bee gut	Egypt	JAHLTK000000000.1	2,7289,28	Zaghloul and Halfawy, 2023
NRRL_B-2354	Milk and dairy utensils	USA	CP004063	2,635,572	Kopit et al. 2014
Gr17	Fermented whole fish product suan yu	China	CP033376.1	2,588,149	Wang et al. 2018
HY07	Chinese sausages	China	CP032308.1	2,585,631	Duan et al. 2018
SRCM103341	Food	South Korea	CP035136.1	2,581,021	Cho et al. 2019
T110	Commercial probiotic product	India	CP006030	2 ,693, 877	Natarajan and Madasamy, 2013
SP15	Natural spring water	Pakistan	RDQA00000000)	2,783,033	Azis et al. 2019
17OM39	Human gut	India	LWHF00000000	2,696,332	Ghattargi et al. 2028
DO	Clinical isolated	USA	CP003583	2,698,137	Qin et al. 2012
Aus0004	Clinical isolated	Australia	CP003351.2	2,952,485	Lam et al. 2012
RBWH1	Clinical isolated	Australia	CP033206.1	2,876,110	Bohlmann et al. 2018
2014-VREF-268	Clinical isolated	Korea	CP019992.1	2,990,310	Kim et al. 2020

*All accession numbers are from GenBank
