# Supplementary material for: Genomic and phenotypic characterization of a honey-derived Enterococcus faecium isolate from the Brazilian semiarid region
Source: Mol Biol Rep. 2026 Nov 1;53(1):1875. doi: 10.1007/s11033-026-13025-w (PMC13724667; doi:10.1007/s11033-026-13025-w)
Supplement: Supplementary file 2 [file 11033_2026_13025_MOESM2_ESM.docx]

***Supplementary material***

**Genomic and Phenotypic Characterization of a Honey-Derived *Enterococcus faecium* Isolate from the Brazilian Semiarid Region**

Dayane da Silva Santos^1#^, Allyson Andrade Mendonça^1#^, Maria Cecília de Lima e Silva^1^, Marcia Maria Camargo de Morais^2^, Danilo Elias Xavier^3^, José Carlos da Rocha Neto^3^, Rafael Barros de Souza^2^ and Marcos Antônio de Morais Júnior ^1*^

^1^Department of Genetics, Federal University of Pernambuco, Recife-Brazil

^2^Institute of Biological Sciences, University of Pernambuco, Recife-Brazil

^3^Department of Microbiology, Institute Aggeu Magalhães, FIOCRUZ, Recife-Brazil

^#^ These authors contributed equally to this work

^*^Author for correspondence:

Marcos Antonio de Morais Junior

Departamento de Genética, Centro de Biociências, Universidade Federal de Pernambuco. Av. Prof. Moraes Rego, 1235, Cidade Universitária, Recife, Pernambuco, 50670-901, Brazil

ORCID: 0000-0002-9727-0300 E-mail: marcos.moraisjr@ufpe.br

**Supplementary Table S1**. Bacterial strains used in this study including GenBank accession number

| **Strain** | **Source** | **Country** | **Accession number** | **Genome length (pb)** | **Reference** |
| --- | --- | --- | --- | --- | --- |
| EFBI58 | Honey | Brazil | JCAGTB010000000 | 2,457,210 | This study |
| Am1 | Bee gut – larvae | Egypt | JAHLTJ000000000 | 2,732,750 | Zaghloul et al. 2022 |
| Bee9 | Bee gut – Adult | Egypt | JAHLXG000000000 | 2,734,007 | Zaghloul et al. 2022 |
| SM21 | Bee gut | Argentina | NZSDXT00000000 | 2,544,244 | Audisio, 2019 |
| H7 | Bee gut | China | CP083179 | 2,529,646 | Du et al. 2021 |
| Am5 | Bee gut | Egypt | JAHLTK000000000.1 | 2,7289,28 | Zaghloul and Halfawy, 2023 |
| NRRL_B-2354 | Milk and dairy utensils | USA | CP004063 | 2,635,572 | Kopit et al. 2014 |
| Gr17 | fermented whole fish product suan yu | China | CP033376.1 | 2,588,149 | Wang et al. 2018 |
| HY07 | Chinese sausages | China | CP032308.1 | 2,585,631 | Duan et al. 2018 |
| SRCM103341 | Food | South Korea | CP035136.1 | 2,581,021 | Cho et al. 2019 |
| T110 | Commercial probiotic product | India | CP006030 | 2 ,693, 877 | Natarajan and Madasamy, 2013 |
| SP15 | Natural spring water | Pakistan | RDQA00000000) | 2,783,033 | Azis et al. 2019 |
| 17OM39 | Human gut | India | LWHF00000000 | 2,696,332 | Ghattargi et al. 2028 |
| DO | Clinical isolated | USA | [CP003583](https://www.ncbi.nlm.nih.gov/nuccore/CP003583) | 2,698,137 | Qin et al. 2012 |
| Aus0004 | Clinical isolated | Australia | CP003351.2 | 2,952,485 | Lam et al. 2012 |
| RBWH1 | Clinical isolated | Australia | CP033206.1 | 2,876,110 | Bohlmann et al. 2018 |
| 2014-VREF-268 | Clinical isolated | Korea | CP019992.1 | 2,990,310 | Kim et al. 2020 |
| Com12 | Feces | USA | GCA_000157635.1 | 2,700,000 | Feldgarden et al.(2014) |
| EnGen007 | Pig | USA | GCF_000321845.1 | 2,700,000 | Earl et al. (2013) |

*All accession numbers are from GenBank.

**Supplementary Table S2.** Quality parameters for the genome sequencing of *Enterococcus faecium* EFBI58.

| Feature / Metric | Value |
| --- | --- |
| Organism | *Enterococcus faecium* strain EFBI58 |
| Source | Honey (Bee-associated environment) |
| NCBI Accession Number | JCAGTB010000000 |
| Total Assembly Length | 2,457,210 bp |
| G+C Content | 38.12% |
| Number of Contigs | 134 |
| Average Sequencing Depth | 78.24× |
| Genome Coverage (≥10×) | 99.99% |
| Genome Coverage (≥30×) | 99.95% |
| N50 Value | 72,922 bp |
| L50 Value | 11 |
| N90 Value | 18,335 bp |
| Mean Contig Length | 18,337 bp |
| Median Contig Length | 2,056 bp |

**Supplementary Table S3.** Average Nucleotide Identity and alignment data (%) for *Enterococcus faecium* EFBI58 compared with *E. faecium* strains from clades A1, A2, and B.

|  |  |  | **EFBI 58** |  |  |
| --- | --- | --- | --- | --- | --- |
|  | Genome | ANib [%] | Alignment [%] | Alignment [bp] | Total [pb] |
| **Clade A1** | DO | 97,69 | 71,51 | 2037007 | 2848380 |
| **Clade A1** | Aus0004 | 98,40 | 74,50 | 2199544 | 2952485 |
| **Clade A1** | RbWH1 | 98,35 | 75,51 | 2171646 | 2876110 |
| **Clade A1** | 2014-VREF | 98,18 | 74,24 | 2219947 | 2990310 |
| Clade **A2** | EnGen007 | 98,58 | 84,24 | 2269469 | 2694048 |
|  | ATCC 8459 | 98,43 | 80,70 | 2275667 | 2820063 |
|  | SM21 | 98,77 | 84,10 | 2139729 | 2544244 |
|  | Bee9 | 98,89 | 84,05 | 2297867 | 2734007 |
|  | Am1 | 98,84 | 84,51 | 2309116 | 2732349 |
|  | Am5 | 98,82 | 84,62 | 2309299 | 2728928 |
| **Clade B** | Com12 | 94,25 | 76,32 | 2071120 | 2713738 |
|  | SP15 | 94,62 | 74,86 | 2083401 | 2783033 |
|  | Hy07 | 94,44 | 78,27 | 2023686 | 2585631 |
|  | Gr17 | 94,63 | 77,07 | 1994736 | 2588149 |
|  | 17OM39 | 94,60 | 73,11 | 2076471 | 2840201 |
|  | T110 | 94,51 | 75,47 | 2033063 | 2693877 |

**Supplementary Figure S1.** Circular comparative genome map of *Enterococcus faecium* strain EFBI58 (2,457,210 bp) aligned with the reference strain *E. faecium* DO.

**Supplementary Figure S2.** UpSetplot showing the distribution of shared and unique coding sequences (CDSs) among *Enterococcus faecium* genomes. The upper bar chart represents the cardinality of each gene set, indicating the number of CDSs shared among the corresponding genome combinations. The lower matrix displays the presence/absence pattern of CDSs across the analyzed genomes, with black dots and connecting lines indicating shared gene clusters. The horizontal bar chart on the left shows the total number of CDSs identified in each genome. The largest intersection represents the conserved core gene set shared among the analyzed genomes, whereas smaller intersections correspond to accessory genes present in specific genome combinations.

**Supplementary Figure S3.** Survival of Enterococcus faecium strain EFBI58 under simulated gastrointestinal conditions. Cell viability (log CFU/mL) was monitored during exposure to simulated gastric fluid (0–2 h) and simulated intestinal fluid (2–6 h).

**Supplementary figure S4.** Circular map of the prophage identified in the genome of *Enterococcus faecium* EFBI58 isolated from honey. The outer ring shows predicted coding sequences colored according to their functional categories, including lysis, replication, regulation, assembly, packaging, integration, infection, miscellaneous functions, and hypothetical proteins. The inner rings represent GC content and GC skew. Genes encoding structural proteins, terminases, portal proteins, integrases, and other phage-related functions were identified, supporting the classification of this region as an intact prophage.

**Supplementary Figure S5.** Phylogenetic analysis of bacteriophages associated with honey-derived *Enterococcus faecium* isolates. The circular phylogenetic tree illustrates the relationship among phage sequences identified in *E. faecium* strains isolated from honey. Phages are grouped into clusters according to genomic similarity, as indicated by the colored outer ring. The branch lengths represent genetic distances, and the distance to the parent node indicates the divergence among phage genomes.
